# Detection of thyroglobulin in fine-needle aspiration for diagnosis of metastatic lateral cervical lymph nodes in papillary thyroid carcinoma: A retrospective study

**DOI:** 10.3389/fonc.2022.909723

**Published:** 2022-09-20

**Authors:** Yuxuan Wang, Yuansheng Duan, Hong Li, Kai Yue, Jin Liu, Qingchuan Lai, Mengqian Zhou, Beibei Ye, Yue Wu, Jiajia Zhu, Peng Chen, Chao Jing, Yansheng Wu, Xudong Wang

**Affiliations:** Department of Maxillofacial and Otorhinolaryngology Oncology, Key Laboratory of Cancer Prevention and Therapy, Tianjin Medical University Cancer Institute and Hospital, Tianjin Cancer Institute, National Clinical Research Center of Cancer, Tianjin, China

**Keywords:** papillary thyroid carcinoma, thyroglobulin, fine need aspiration, lymph nodes, metastasis

## Abstract

**Objective:**

We analysed the diagnostic performance of thyroglobulin in fine-needle aspiration (FNA-Tg) in the suspicious lateral cervical lymph nodes (CLNs) in patients with papillary thyroid cancer (PTC), proposed the best cutoff value and discussed the factors that may affect the diagnostic value of FNA-Tg.

**Methods:**

In the present study, a retrospective analysis of 403 patients with PTC with 448 suspected lateral CLNs metastasis from October 2019 to May 2021 was performed. The cutoff value according to the receiver operating characteristic (ROC) curve was determined, and the Wilcoxon rank-sum test was used to evaluate the correlation between FNA-Tg and factors.

**Results:**

According to the ROC curve, the cutoff value of FNA-Tg was 3.69 ng/ml (sensitivity, 92.48%; specificity, 75.00%). Patients who underwent total thyroidectomy were excluded. Compared with US and FNAC, the diagnostic performance of FNA-Tg was the greatest, especially for small CLNs (diameter ≤ 1 cm), cystic CLNs, and patients with Hashimoto’s thyroiditis (HT). Moreover, FNA-Tg levels were correlated with the presence of HT (*p* = 0.003), the anti-thyroglobulin antibody (Tg-Ab) (*p* < 0.001), the ratio of metastatic lateral CLNs (*p* = 0.004) and Tg assay kits (*p* < 0.001).

**Conclusions:**

FNA-Tg measurement is sensitive enough for diagnosing lateral CLN metastases from PTC, but its diagnostic value is compromised by a number of factors.

## Introduction

Papillary thyroid carcinoma (PTC) accounts for 90% of thyroid cancer diagnoses (1) and has the best prognosis among them. However, metastasis to the cervical lymph nodes (CLNs) occurs in 20–50% of cases, resulting in recurrence (2), and accurate CLN examination is critical to treatment strategy and patient outcome. Ultrasound (US) is widely available, convenient and economical with a high degree of sensitivity (3) but still fails to recognize CLNs of diameter less than 1 cm (4). In addition, the diagnostic value of US is affected by the presence of lymphocytes, variable necrosis and poor epithelial cellularity (5). US-guided fine-needle aspiration of cytology (FNAC) has demonstrated high specificity in detecting solid CLNs. Previous studies have assigned FNAC sensitivity rates of 66–95% and specificity of 40–100% (6–9). However, FNAC detection of CLNs leads to the identification of cystic components, false-negative results and non-diagnostic interpretations due to the lack of epithelial tissue (10, 11). Pacini et al. first proposed the measurement of thyroglobulin in fine-needle aspiration (FNA-Tg) washout fluids in 1992 (12), giving a sensitivity and specificity of 100% and hugely advancing the assessment of CLNs. Both the American Thyroid Association (ATA) and the European Thyroid Association (ETA) guidelines strongly recommend the use of FNA-Tg to evaluate CLNs prior to surgery, especially for patients with suspected recurrence after total thyroidectomy (13, 14). However, no uniform standard for the cutoff value of FNA-Tg for diagnosis of CLN metastasis in PTC exists. The presence or absence of thyroid glands (15), levels of serum-Tg (s-Tg) (16) and levels of anti-thyroglobulin antibody (Tg-Ab) may all affect the diagnostic performance of FNA-Tg (17). Thus, the utility of FNA-Tg, remains controversial.

The aim of the current study was to identify the optimal FNA-Tg cutoff for diagnosis of lateral CLN metastasis in PTC patients. Sensitivity and specificity of US, FNAC and FNA-Tg were examined to compare diagnostic performances. Factors affecting the diagnostic value of FNA-Tg were analyzed to give a reliable basis for clinical diagnosis and application.

## Materials and methods

### Experimental subjects

A retrospective analysis of 448 lateral CLNs from 403 patients who underwent unilateral or bilateral therapeutic lateral neck dissection with or without thyroidectomy due to recurrence/metastases of PTC between October 2019 and May 2021 at Tianjin Medical University Cancer Institute and Hospital was conducted. Eleven patients underwent unilateral lateral CLN dissection without thyroidectomy due to suspicious recurrence in the CLNs only and had previously received unilateral thyroidectomy. Patients with histories of total thyroidectomy surgery were excluded due to the small sample size and difficulty of obtaining a significant result. Final CLN diagnoses were based on postoperative histopathology.

Ethical approval was granted by Tianjin Cancer Hospital Ethics Committee. No patient had a contraindication to puncture and written informed consent was obtained from all participants prior to the operation.

### FNAC and FNA-Tg

All patients underwent US neck examination and suspected malignant lymph nodes were identified according to the following characteristics: calcification, loss of the hilar, focal or diffuse hyperechoic changes, cystic change round shape and abnormal blood flow signal. Suspected malignant lateral CLNs were aspirated 3-5 times each by experienced US experts using a 10 ml syringe carrying a 22-gauge needle under high-frequency US real-time localization. Aspirated tissue from each CLN was smeared onto two to three slides, fixed with 95% ethanol and stained with Papanicolaou staining for cytological diagnosis by an experienced pathologist.

Remaining aspirates on the puncture needle were washed with 1 ml of 0.9% normal saline, collected and tested for Tg concentration over time by chemiluminescence immunoassay (ECLIA), using a commercially available kit (Elecsys Tg II). The measuring range was 0.04–500 ng/ml.

### Statistics

Statistical Package for Social Science (SPSS) professional software version 21.0 was used. Taking histopathological results as the gold standard, ROC curves of FNA-TG were drawn and the area under the ROC curve (AUC) calculated. Sensitivity, specificity, positive predictive value (PPV), false predictive value (NPV) and accuracy were calculated to compare the diagnostic value of methods, according to the following formulae: Sensitivity=True positive/(True positive+ False negative); Specificity=True false/(True negative+ False positive); PPV=True positive/(True positive+ False positive); NPV=Ture false/(True negative+ False negative); Accuracy=(True negative+ True positive)/Total. A Wilcoxon rank-sum test was used to evaluate the correlation between FNA-Tg and s-Tg, TPO-Ab and Tg-Ab. A p-value of <0.05 was regarded as statistically significant.

## Results

### Clinical characteristics and lateral CLNs

A total of 403 patients (129 males and 274 females) with 448 lateral CLNs of median size 1.2 cm (range: 0.4–4.9 cm) were recruited over a 20-month period. Patient mean age was 41.37 years (range: 8–75 years). A total of 398 lateral CLNs were suspected to be malignant from US results and the remaining 50 considered inflammatory. 338 (81.06%) CLNs were suspected of being malignant after FNAC and 38 were benign (9.11%). 72 (16.07%) CLNs could not be accurately characterized due to insufficient samples or inefficient data. The median size of CLNs undiagnosed by FNAC was 1.1 cm and FNA-Tg analysis gave an accuracy of 83.3%, indicating the utility of this measurement in overcoming the drawbacks of FNAC. Final histopathological diagnosis identified 412 (91.96%) of CLNs as metastatic and 36 (8.04%) as benign.

### Differences between metastatic and benign lateral CLNs

**Figure 1:**
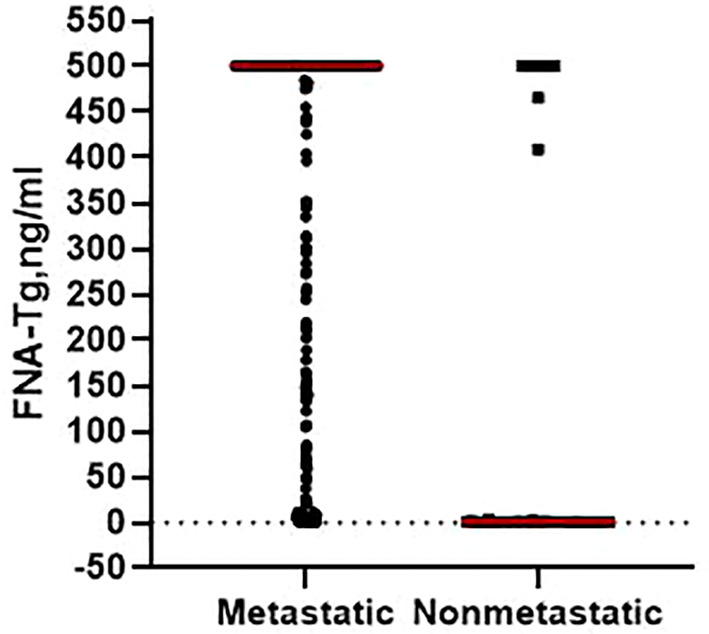
The level of FNA-Tg in metastatic and non-metastatic lateral CLNs.

Post-surgery histopathological analysis allowed the CLNs to be divided into metastatic and benign groups. Mean patient age was younger in the metastatic group (40.9 ± 12.7 years) than in the benign (46.5 ± 12.5 years; p = 0.013). Median CLN size in the metastatic group (1.3 cm) was higher than in the benign group (1.0 cm; p = 0.004). Percentage metastatic lateral CLNs was significantly higher in men (94.59%) than in women (90.67%; p = 0.015) and metastatic CLNs had higher median levels of FNA-Tg (p < 0.001; **Figure 1**) and s-Tg (p = 0.03) than benign CLNs. Other indicators were not significantly different between the metastatic and benign groups (**Table 1**).

**Table 1:** Difference between metastasis and benign of lateral CLNs.

	Metastasis (412)	Benign (36)	P value
Age, years	40.9 ± 12.7	46.5 ± 12.5	0.013
Sex			0.015
male	140	8	
female	272	28	
Size of tumor, cm, (median, range)	1.1 (0.1-7.0)	0.9 (0.1-2.5)	0.374
Size of CLNs, cm, (median, range)	1.3 (0.4-4.9)	1.0 (0.4-2.7)	0.004
ETE (presence:absence)	49:325 (15.08%)	5:30 (16.67%)	0.843
HT(presence:absence)	114:260 (43.85%)	10:17 (58.82%)	0.477
s-Tg, ng/ml, (median, range)	24.1 (0.04-510)	15.9 (0.14-90)	0.030
TSH, mIU/L, (median, range)	2.1 (0.01-49.6)	2.2 (0.03-6.4)	0.674
Tg-Ab, IU/ml, (median, range)	13.1 (0.92-4000)	14.0 (4.79-4000)	0.178
TPO-Ab, IU/ml, (median, range)	9.0 (0.25-1022)	9.0 (9-375)	0.058
FNA-Tg, ng/ml, (median, range)	500.0 (0.04-500)	0.45 (0.04-500)	<0.001

CLNs, Cervical Lymph Nodes; ETE, Extrathyroidal extension; HT, Hashimoto’s thyroiditis; s-Tg, Serum thyroglobulin; FNA-Tg, The measurement of thyroglobulin on fine-needle aspiration; Tg-Ab, anti-thyroglobulin antibody; TPO-Ab, anti-peroxidase antibody.

### Cutoff values of FNA-Tg for malignant lateral CLNs

**Figure 2:**
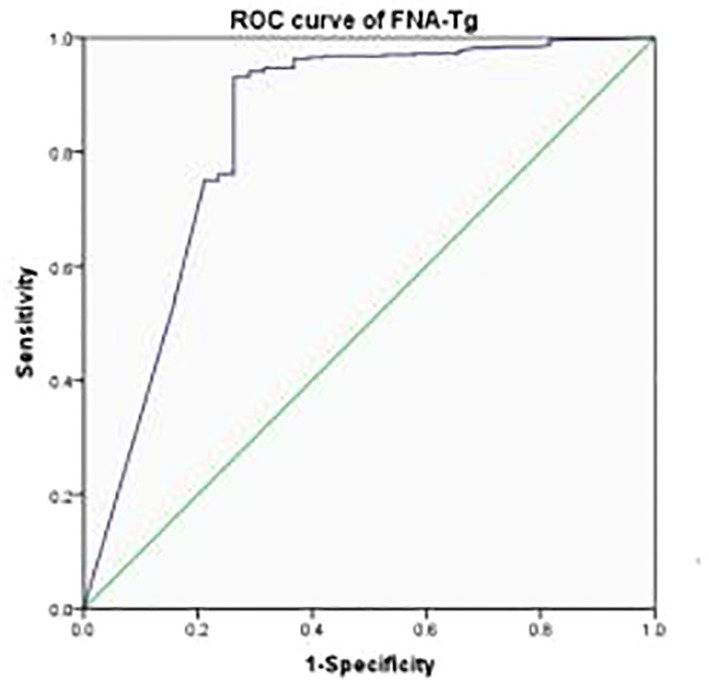
The ROC curve of FNA-Tg according to definite status of the 448 lateral CLNs.

The most appropriate cutoff value for diagnosing metastatic lateral CLNs in PTC was estimated from the ROC curve to be 3.69 ng/ml with an AUC of 0.839 (**Figure 2**) and sensitivity, specificity and accuracy of 92.48%, 75.00% and 91.07%, respectively. A total of 412 CLNs were histopathologically defined as malignant and 381 (92.48%) had levels of FNA-Tg in excess of 3.69 ng/ml. However, FNA-Tg analysis of the 48 benign CLNs showed only 27 (56.25%) to have low levels. Evaluation of the diagnostic performance of multiple FNA-Tg levels (0.04, 0.1, 0.5, 3.69, 5, 10, 20 and 50) showed that FNA-Tg sensitivity decreased but specificity increased with an increase in the diagnostic threshold (**Table 2**).

**Table 2:** Diagnostic value in different cutoff values of FNA-Tg.

Different cutoff values, ng/ml	Sensitivity (%)	Specificity (%)	PPV (%)	NPV (%)	Accuracy (%)
FNA-Tg (0.04)	99.03	25.00	93.79	69.23	93.08
FNA-Tg (0.1)	97.09	30.56	94.12	47.83	91.74
FNA-Tg (0.5)	95.87	52.78	95.87	52.78	92.41
FNA-Tg (1)	95.39	66.67	97.04	55.81	93.08
FNA-Tg (3.69, ROC)	92.48	75.00	97.69	46.55	91.07
FNA-Tg (5)	91.50	75.00	97.67	43.55	90.18
FNA-Tg (10)	90.29	75.00	97.64	40.30	89.06
FNA-Tg (20)	88.35	75.00	97.59	36.00	87.28
FNA-Tg (50)	87.14	75.00	97.55	38.57	86.16
FNA-Tg/s-Tg>1	85.47	77.78	95.00	34.15	84.82

FNA-Tg, The measurement of thyroglobulin on the fine-needle aspiration; PPV, Positive predictive value; NPV, Negative predictive value.

The presence of thyroid glands may cause false positive results and the ratio of FNA-Tg and s-Tg were used as the cutoff value to eliminate the interference of s-Tg. FNA-Tg/s-Tg >1 gave the highest specificity but lower sensitivity (**Table 2**). We found a higher cutoff value for FNA-Tg (3.69 ng/ml) than that previously reported in the literature (>1 ng/ml). A total of 15 lymph nodes with FNA-Tg values between 1-3.69 ng/ml were found during the current study (**Table 3**). A comparison of the 1 ng/ml and 3.69 ng/ml cutoff values revealed improved specificity at the expense of lower sensitivity. In summary, we find a cutoff value of 3.69 ng/ml FNA-Tg to be the most appropriate because of its relatively high sensitivity and specificity.

**Table 3:** Cases of CLNs with FNA-Tg between 1-3.69 ng/ml.

Number		15
FNAC	Positive	8
	Negative	5
	Inefficient	2
Size of CLNs, cm, (median, range)		1.3 (0.8-2.6)
Pathology	Metastasis	12
	Benign	3

CLNs, Cervical Lymph Nodes; FNAC, Fine needle-aspiration cytology.

**Figure 3:**
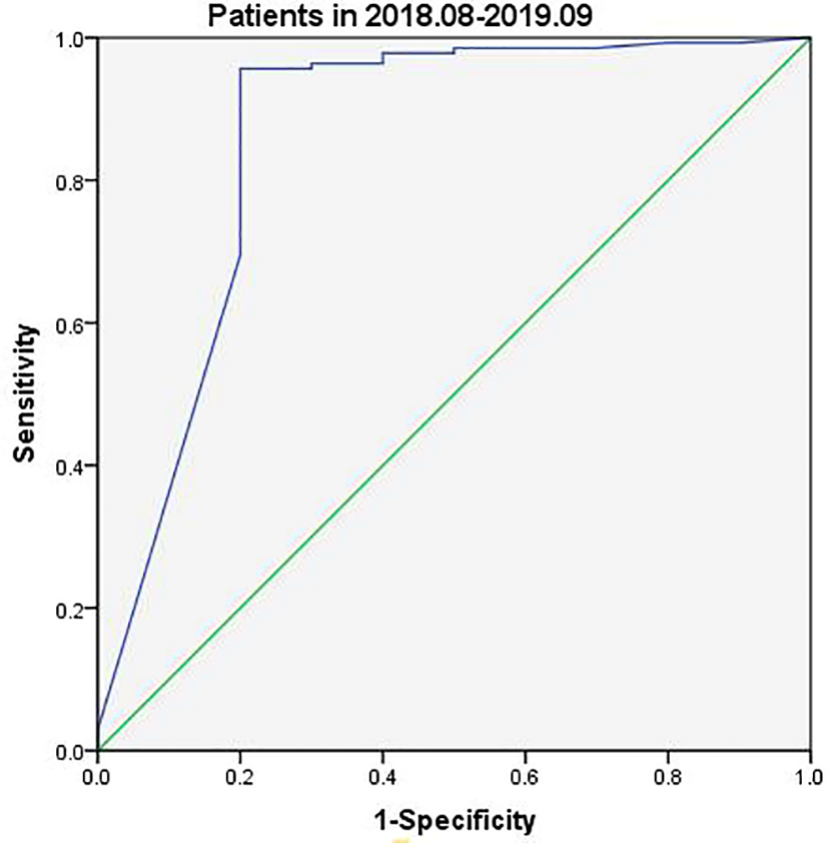
The ROC curves of FNA-Tg in different assay kits of Tg (Patients in 2018.08-2019.09).

**Figure 4:**
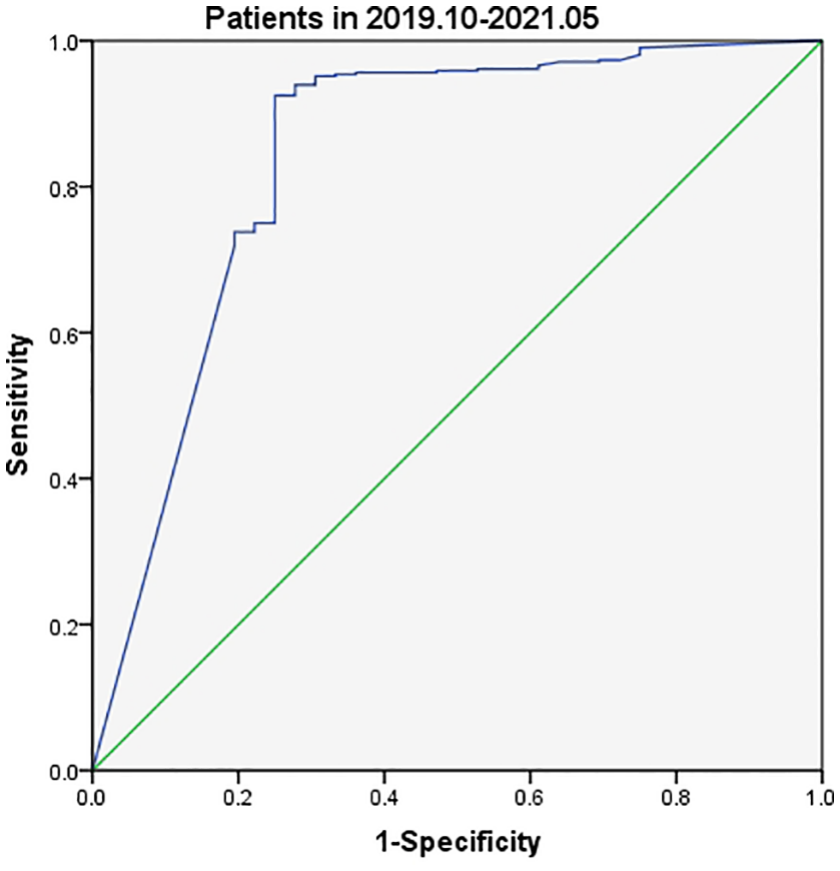
The ROC curves of FNA-Tg in different assay kits of Tg (Patients in 2019.10-2021.06).

Tg assay kits were changed during the current study with Beckman Access Tg 2 assay (functional sensitivity: 0.1–482 ng/ml) being used between August 2018 and September 2019 and Elecsys Tg 2 assay (functional sensitivity: 0.04–500 ng/ml) between October 2019 and May 2021. Analysis of data collected between August 2018 and September 2019 indicated slightly different FNA-Tg cutoff values derived from the use of the two kits: 3.15 ng/ml vs. 3.69 ng/ml (**Figures 3**, **4**). Median FNA-Tg level was lower when measured by the Beckman Access Tg 2 assay (482 ng/ml) than by the Elecsys Tg 2 assay (500 ng/ml; p < 0.001).

### Comparison of diagnostic value of US, FNAC and FNA-Tg

Characteristics of suspicious CLNs from US scans included cystic change, calcification or rounded rather than elongated shape, abnormal blood flow, focal or diffuse hyperechoic changes, absence of hilum and irregular margins (18). Sensitivity, specificity, PPV and NPV of US were 90.39%, 30.56%, 93.62% and 22.00%, respectively. PTC-like nuclear features in the follicular cells of LNs, such as nuclear enlargement and overlapping, irregular membrane, grooves and pseudoinclusion bodies with obvious nuclear clearance, led to identification of PTC metastasis by FNAC (19). FNAC showed a sensitivity of 92.39% and a specificity of 52.38% across 448 lateral CLNs. Combining US, FNAC and FNA-Tg gave the highest sensitivity (98.50%) and accuracy (91.53%) but the lowest specificity (13.89%; **Table 4**).

**Table 4:** Comparison of diagnostic value between US, FNAC and FNA-Tg.

Diagnostic methods	Sensitivity (%)	Specificity (%)	PPV (%)	NPV (%)	Accuracy (%)	AUC(95%CI)
FNA-Tg(3.69, ROC)	92.48	75.00	97.69	46.55	91.07	0.839 (0.754,0.925)
FNAC	92.39	52.38	97.04	28.95	90.16	0.540 (0.414,0.665)
US	90.39	30.56	93.62	22.00	85.52	0.616 (0.522,0.710)
FNAC+US	97.24	20.00	95.09	31.25	92.67	0.787 (0.688,0885)
FNAC+FNA-Tg(3.69)	94.34	60.71	97.09	43.59	92.07	0.815 (0.712,0.918)
US+FNA-Tg(3.69)	98.04	27.78	93.90	55.56	92.34	0.835 (0.753.0.918)
FNAC+US+FNA-Tg(3.69)	98.50	13.89	92.72	45.45	91.53	0.823 (0.725,0.922)

PPV, Positive predictive value; NPV, Negative predictive value; AUC, Area under the curve; US, Ultrasound; FNAC, Fine needle-aspiration cytology; FNA-Tg, The measurement of thyroglobulin on fine-needle aspiration.

For 72 CLNs that could not be diagnosed by FNAC, the accuracy of FNA-Tg was up to 83.3%. FNAC specificity and PPV were up to 100% for 55 cystic CLNs identified by US but accuracy (90.38%) and sensitivity (90.00%) were lower than that of FNA-Tg (94.55% and 98.08%). The accuracy of US (85.87%) and FNAC (88.31%) for the 112 small lymph nodes with diameters less than or equal to 1 cm indicated by US was lower than that of FNA-Tg (92.47%; **Table 5**). The diagnostic value of FNA-Tg was higher for 124 lateral CLNs with HT than that of FNAC and US, regardless of sensitivity, specificity, PPV, NPV or accuracy.

**Table 5:** Different methods to diagnose small CLNs.

Methods	Sensitivity (%)	Specificity (%)	PPV (%)	NPV (%)	Accuracy (%)
FNAC	88.31	50.00	91.89	40.00	83.15
US	85.87	42.86	86.81	40.91	77.78
FNA-Tg	92.47	76.19	94.51	69.57	89.47

PPV, Positive predictive value; NPV, Negative predictive value; US, Ultrasound; FNAC, Fine needle-aspiration cytology; FNA-Tg, The measurement of thyroglobulin on fine-needle aspiration.

### Factors influencing FNA-Tg diagnostic efficiency

Level of Tg-Ab (p < 0.001), presence of HT (p = 0.003), lymph node ratio (LNR) (p < 0.001) and Tg assay kits (p < 0.001) were all found to be related to the level of FNA-Tg (**Table 6**).

**Table 6:** Factors may influence the level of FNA-Tg.

Factors	p Value
Year	0.23
Sex	0.062
s-Tg	0.104
TSH	0.368
Tg-Ab	<0.001
TPO-Ab	0.112
CT	0.099
Size of CLNs	0.071
Characteristics of CLNs (cystic or solid)	0.54
LNR	<0.001
ETE	0.768
Subtypes of PTC	0.885
BRAF	0.365
TNM	0.549
HT	0.003
Types of Tg assay kits	<0.001

CLNs, Cervical Lymph Nodes; ETE, Extrathyroidal extension; HT, Hashimoto’s thyroiditis; s-Tg, Serum thyroglobulin; FNA-Tg, The measurement of thyroglobulin on fine-needle aspiration; Tg-Ab, anti-thyroglobulin antibody; TPO-Ab, anti-peroxidase antibody; CT, calcitonin; LNR, the lymph node ratio.

Samples were divided into Tg-Ab positive, Tg-Ab negative and Hashimoto’s thyroiditis (HT) positive and HT negative groups, according to the level of antibodies and the HT status. Samples were defined as Tg-Ab positive if levels were greater than or equal to 115 IU/ml. Median Tg-Ab levels were similar in the positive and negative groups but the interquartile range (IQR) was higher in the former (500–6.675) than in the latter (500–441), indicating more concentrated FNA-Tg at 500 ng/ml in patients with negative Tg-Ab. ROC curves demonstrated a lower FNA-Tg cutoff value of in the Tg-Ab-positive group (1.525 ng/ml) than in the negative group (4.165 ng/ml). Similarly, equivalent median FNA-Tg levels were found in the HT-positive and negative groups but the IQR was significantly different (HT positive: 500–104.5 and HT negative: 500–470, p = 0.003). The degree of FNA-Tg dispersion was small in the HT-negative group and the cutoff value in patients with HT (1.525 ng/ml) was lower than in patients without HT (4.165 ng/ml). The LNR was determined by dividing the number of invaded CLNs by the total number of removed CLNs and was found to be related to FNA-Tg level (p < 0.001). Above a cutoff value equal to the median level of LNR (0.16), FNA-Tg was more concentrated at 500 ng/ml.

None of s-Tg level (p = 0.104), CLN characteristics (cystic or solid, p = 0.54) or CLN size (p = 0.071) affected FNA-Tg level. Postoperative histopathological results divided PTC samples into classic, follicular, Warthin tumor-like and diffuse sclerosing types. Levels of FNA-Tg did not vary according to these different subtypes (p = 0.885).

## Discussion

Recent evidence has accumulated to suggest that the diagnostic value of FNA-Tg for CLN metastasis was significantly better than that of US and FNAC. A recent meta-analysis of 2257 patients with 2786 suspicious CLNs gave a hierarchy of diagnostic value as follows: a combination of FNAC and FNA-Tg > FNA-Tg > FNAC (20). The current research affirmed the clinical value of FNA-Tg and achievement of the best diagnostic performance by FNA-Tg, FNAC and US. Se Jeong Jeon et al. concluded that FNA-Tg gave superior diagnosis of metastasis in small CLNs (≤5 mm in minimal diameter) (21). Such small nodes are relatively difficult to aspirate and sample by FNAC, complicating the interpretation of cytology results (5). The current study demonstrated a diagnostic accuracy of FNA-Tg of 92.47% for small CLNs (diameter < 1 cm), higher than that of US (85.87%) and FNAC (88.37%). Metastatic CLNs (median, 1.3cm) were found to be bigger than benign CLNs (median, 1.0 cm) and had higher FNA-Tg levels (median: 500 ng/ml vs 0.45 ng/ml) but CLN size was not directly related to FNA-Tg level. We consider FNA-Tg, to be a qualitative rather than quantitative detection indicator. Cystic changes in CLNs were seen as malignant signs by US but presented a challenge for FNAC because of scant cellularity (22). Analysis of the diagnostic value of FNAC and FNA-Tg for cystic CLNs gave higher sensitivity (98.08%) but lower specificity (33.33%) for FNA-Tg compared with FNAC (90.00% and 100%). We strongly recommend the combination of FNA-Tg and FNAC for diagnosis and analysis of cystic CLNs. HT has been frequently associated with PTC occurrence and the interference of large numbers of lymphocytes complicates judgment of CLN characteristics by US and FNAC. Coexistence of PTC and HT among the current cohort was 27.68% (124/448). We found a superior diagnostic performance of FNA-Tg over FNAC for HT patients and a combination of the two methods produce the best results of all.

From the first proposal of FNA-Tg utility in 1992 until the present, no standard for the diagnostic threshold of FNA-Tg has been agreed. A meta-analysis of 24 studies and 2865 LNs showed a range of FNA-Tg cutoff values from 0.2 ng/ml to 50 ng/ml in patients with or without thyroid glands (23) and a cutoff of 1 ng/ml was frequently selected (24, 25). Variations in patient cohorts, different types of washout fluids, different Tg assay kits and the potential impact of some clinical biochemical indicators may all contribute to producing the wide range of values. Central CLNs are subject to interference by thyroid glands and the trachea (26, 27), leading to false-positive results, and we chose to include only lateral CLNs in the current study. The cutoff value of 3.69 ng/ml derived from ROC curve (AUC=0.839) analysis of the present cohort is in agreement with most current research (16, 28) but is lower than some recent values (12, 29). Some studies have proposed that functional assay sensitivity, lower in the early stages, affects cutoff values, explaining the progressive decrease in proposed cutoff values (30). Similar findings were supported by the current study due to the impact of variable Tg assay kits.

Baskin (2004) proposed that serum Tg-Ab had no effect on diagnostic performance because intracellular Tg was not exposed to circulating Tg-Ab (31). In addition, the concentration of FNA-Tg in metastatic CLNs was suggested to be much higher than that of Tg-Ab, leading to saturation of Tg-Ab binding sites and overcoming the interference (19). However, the study of 207 patients with 263 CLNs by Min Ji Jeon et al. demonstrated lower FNA-Tg levels in CLNs from serum Tg-Ab-positive patients than in those from Tg-Ab-negative patients (p = 0.001) (17). We confirm the above findings and recommend that a lower cutoff value of FNA-Tg should be used for patients with a high level of Tg-Ab. We speculated that the presence of Tg-Ab in CLNs from patients with high serum Tg-Ab may bind to Tg within the CLN, resulting in decreased detection of FNA-Tg. We aim to measure Tg-Ab within CLN tissue in order to confirm or refute our suspicions.

HT may interfere with FNA-Tg levels. The condition is often accompanied by increased Tg-Ab due to a complex autoimmune and inflammatory response which destroys normal follicular cells in the thyroid gland and precipitates the occurrence of tumors (32). Interestingly, patients with higher levels of Tg-Ab or who were positive for HT had similar FNA-Tg cutoff values (1.525 ng/ml) and *vice versa*. The diagnostic performance of FNA-Tg was profoundly affected by the presence of HT. Lower cutoff values of FNA-Tg are advised for HT patients and the combination of FNAC and FNA-Tg recommended for diagnosis of metastatic CLNs. In view of these findings, we recommend that all patients prescribed CLN FNA-Tg measurements also have bloodwork for serum Tg-Ab levels and TPO-Ab to identify HT.

s-Tg has been found to cause false positive FNA-Tg results in many previous studies (9, 33). However, the current study found that s-Tg levels were not related to FNA-Tg levels (p=0.104),in agreement with Anne-Laure Borel et al. (34). Contamination by s-Tg during puncture is likely to be negligible due to extremely high FNA-Tg concentrations (usually higher than the maximum value of the detection range).

LNR was related to FNA-Tg level but when FNA-Tg was very high in the lower internal jugular chain (e.g., 500 ng/ml), CLNs were shown by histopathological analysis to occur in areas other than the lower internal jugular chain. Thus, FNA-Tg measurement does not aid the determination of the extent and area of metastasis. We recommend that the surgeons base treatment decisions on intraoperative findings in combination with preoperative evaluations. FNA-Tg is affected by diverse factors, all of which require consideration by treating physicians.

We acknowledge some limitations to the current study. Patients with histories of total thyroidectomy surgery (only 39) were excluded due to the paucity of data. However, thyroid glands may release Tg, causing contamination during puncture and false positive results. This may result a higher cutoff value in the current study compared with others and we aim to collect more data from patients who have undergone total thyroidectomy to improve cutoff value accuracy. Many patients with an extremely low level of FNA-Tg (such as 0.04 ng/ml) did not have surgical treatment. We were unable to judge whether the CLNs were benign or malignant in these patients. In addition, it has been suggested that FNA causes a surge in s-Tg, illustrating the importance of measuring s-Tg before FNA. No clear chronological order was observed for the examination of patients in the current study and future research should be avoid interference by these factors. Moreover, many patients showed a lower level of FNA-Tg after puncture of the lower internal jugular chain of the neck and CLNs from this area only were resected. Histopathology confirmed the absence of metastasis in this area but whether metastases were present in other areas was unknown. In addition, the current study population was relatively young (mean age: 41.37), with small tumor size (median: 1.1cm) and a high percentage of males (33.04%). This may lead to generalizability bias, as young PTC patients tend to have more aggressive disease with higher rates of regional metastasis and extra-thyroid extension. Therefore, this inevitable bias may lead to different results from other studies. Finally, all patients had more than one suspected malignant CLN but only one with obvious signs of metastasis was selected for puncture. Punctured CLNs could not be identified during surgeries but resection range ensured that every suspicious CLN was removed. Had labelling of CLNs been possible during surgery, more reliable results may have been obtained.

## Conclusions

US guided FNA-Tg was an efficient preoperative diagnostic procedure, showing good performance for PTC patients with suspicious lateral CLNs. The technique depends on the availability of experienced cytopathologists, especially for small or cystic CLNs. Optimal diagnostic value was achieved by a combination of US, FNAC and FNA-Tg. Ideal cut-off values for FNA-Tg require further validation. The presence of HT, high Tg-Ab levels and LNR all affected the level and diagnostic value of FNA-Tg. Different Tg assay kits may produce different cutoff values because of the differences in range and functional sensitivity. The clinical value of FNA-Tg continues to be acknowledged but should only be used as an auxiliary diagnostic method and does not determine the range of surgical removal of CLNs.

## Data availability statement

The raw data supporting the conclusions of this article will be made available by the authors, without undue reservation.

## Ethics statement

The study involving human participants were reviewed and approved by Tianjin Cancer Hospital Ethics Committee. All patients had no contraindications to puncture and signed informed consent before the operation. The patients provided written informed consent to participate in this study.

## Author contributions

All authors contributed to the study conception and design. Material preparation, data collection and analysis were performed by YXW, YSD, HL, KY, JL, QCL, MQZ, BBY, YW, CJ, YSW, and XDW. The first draft of the manuscript was written by XDW, YSD, and HL. All authors commented on previous versions of the manuscript. All authors read and approved the final manuscript.

## Acknowledgments

Grateful acknowledgement is made to my supervisor XW who gave me considerable help by means of suggestion, comments and criticism. His encouragement and unwavering support has sustained me through frustration and depression. Without his pushing me ahead, the completion of this review would be impossible. At the same time, I would also like to thank YD for answering my questions and thank for HL giving me help in the revision of the review. Last but not the least, my gratitude also extends to my family who have been assisting, supporting and caring for me all of my life.

## Conflict of interest

The authors declare that the research was conducted in the absence of any commercial or financial relationships that could be construed as a potential conflict of interest.

## Publisher’s note

All claims expressed in this article are solely those of the authors and do not necessarily represent those of their affiliated organizations, or those of the publisher, the editors and the reviewers. Any product that may be evaluated in this article, or claim that may be made by its manufacturer, is not guaranteed or endorsed by the publisher.
